# Pre-existing heterologous immunity to poliovirus vaccination may mitigate severity of hand, food and mouth disease caused by EV71

**Published:** 2011-01-10

**Authors:** Q Leng, C Yang, K Zhu, C Deng, L Zhu, J Wan

**Affiliations:** 1 Unit of Immune Regulation, Institut Pasteur of Shanghai, Chinese Academy of Sciences, Shanghai 200025, PR China

## 

The hand, foot and mouth disease (HFMD) has emerged as a major infectious disease affecting children in China since March, 2008. Over 2.7 millions cases have been reported according to the proceeding of the Ministry of Health of China so far. Majority of patients have mild symptoms such as fever and blisters in the mouth and a skin rash, but some patients develop more severe neurologic symptoms and even die from pulmonary edema due to brainstem infection. Of note, severe cases and fatalities arise dramatically this year. However, what factors cause the current outbreaks and affect the clinical outcome of patients remains unclear. We carried out a retrospective case-control study on Fuyang HFMD outbreaks. We found that the proportions of children who had timely received poliovirus vaccine are 70.9%, 62.4% and 41.7% in health control, HFMD patients without pulmonary edema and HFMD patients with pulmonary edema, respectively. There was a significant difference in the proportion of children who had received the recommended OPV immunization between the group with edema and the controls. In addition, there was also a significant difference between the patients with edema and the one without edema, suggesting that the untimely poliovirus vaccination correlates to the HFMD severity, namely pulmonary edema. Our recent preliminary study in animal model suggests that pre-existing memory CD4 T cells specific for poliovirus can recognize EV71 and may help to mitigate the disease of EV71 infection.

